# Imaging of bone and soft tissue tumours: a case study approach

**DOI:** 10.1038/sj.bjc.6600083

**Published:** 2002-02-01

**Authors:** R Green

**Affiliations:** Department of Radiology, The Royal National Orthopaedic Hospital NHS Trust, Stanmore, Middlesex,UK

## Abstract

*British Journal of Cancer* (2002) **86**, 497–497. DOI: 10.1038/sj/bjc/6600083 www.bjcancer.com

© 2002 The Cancer Research Campaign

## Imaging of bone and soft tissue tumours: a case study approach

T Rand, P Ritschl, S Trattnig, M Breitensaher, H Imhof and D Resnick. Publisher: Springer-Verlag. ISBN 3-540-65096-2. $69.00. 168 pages.

An interesting and wide selection of cases of bone and soft tissue tumours are presented in the following format: On the first page, a brief history is given, then the imaging findings. One then has to turn the page to see the images (both plain film and cross sectional imaging) that have already been described. On the facing page to the images is a list of differential diagnoses based on plain radiography and then based on more advanced imaging findings; each of these is then discussed as to why it enters the differential diagnosis and why it may or may not represent the final diagnosis. One then turns the page to find the final diagnosis, with a short summary of the clinical data relating to the tumour.

The quality of the cases has not been given justice by the layout of the book; this could have been improved by having the imaging and description of the images on facing pages. Unfortunately a lot of the image reproduction is poor. There is no index in which to access specific tumours, and the contents contained purely a list of sites in which the lesions occurred. At the end of the book, lists of recommended reading are given for specific tumours, which are grouped by cell type, although are not referred to in the main body of the text.

The subject matter that is covered is specialized. The book demonstrates a technique for the analysis of bone and soft tissue tumour cases, with limited text covering the pathology of each lesion. This is a book that could be held by a well-funded library as a demonstration of an analytical approach to bone and soft tissue tumours and a method by which a trainee or clinician could test their diagnostic skills.

